# Successful simultaneous stenting of a pulmonary artery and vein in pulmonary vascular stenosis due to silicosis. Case report and literature review

**DOI:** 10.3389/fcvm.2023.1108768

**Published:** 2023-05-09

**Authors:** M. Westhoff, T. Hardebusch, P. Litterst, A. Breithecker, M. Haas, M. Kuniss, T. Neumann, S. Guth, C. B. Wiedenroth

**Affiliations:** ^1^ Department of Pulmonology, Klinik für Pneumologie, Schlaf und Beatmungsmedizin, Lungenklinik Hemer, Zentrum für Pneumologie und Thoraxchirurgie, Hemer, Germany; ^2^ Universität Witten/Herdecke University, Witten, Germany; ^3^ Department of Radiology, Kerckhoff Heart and Thorax Center, Bad Nauheim, Germany; ^4^ Department of Cardiology, Kerckhoff Heart and Thorax Center, Bad Nauheim, Germany; ^5^ Department of Thoracic Surgery, Kerckhoff Heart and Thorax Center, Bad Nauheim, Germany

**Keywords:** mediastinal fibrosis, silicosis, pulmonary artery stenosis, pulmonary vein stenosis, pulmonary hypertension, stenting

## Abstract

A 58-year-old patient was admitted to the emergency department due to severe respiratory insufficiency. Anamnesis revealed that the patient had experienced increasing stress dyspnea for a few months. Upon imaging, an acute pulmonary embolism was excluded, but peribronchial and hilar soft tissue proliferation with compression of central parts of the pulmonary circulation was found. The patient had a history of silicosis. The histology report showed tumor-free lymph node particles with prominent anthracotic pigment and dust depositions without evidence of IgG4-associated disease. The patient was administered steroid therapy and underwent simultaneous stenting of the left interlobular pulmonary artery and the upper right pulmonary vein. As a result, a significant improvement in symptoms and physical performance was achieved. The diagnosis of inflammatory or, in particular, fibrosing mediastinal processes can be challenging and important clinical symptoms must be taken into account, especially if the pulmonary vasculature is involved. In such cases, the possibility of interventional procedures should be examined in addition to drug therapy options.

## 1. Introduction

Inflammatory and especially fibrosing mediastinal processes can lead to significant differential diagnostic challenges and key clinical symptoms, especially if pulmonary vascular involvement is present (1–4). In cases of definitively confirmed pulmonary arterial and/or venous stenosis, it is important to clarify whether interventional procedures are an option in addition to drug therapy (5–7).

The following case description reveals the chameleon-like clinical symptoms of an extremely rare case of simultaneous pulmonary artery (PA) and pulmonary vein (PV) stenosis as a result of silicosis, the considerable challenges in confirming the diagnosis, and the currently existing therapeutic options for the treatment of pulmonary vascular co-involvement in fibrosing mediastinal diseases.

## 2. Case description

In June 2020, a 58-year-old patient with known history of silicosis first presented due to increasing dyspnea under stress. The blood gases in the referring clinic showed hyperventilation and hypoxemia (pH 7.47, pCO_2_ 32 mmHg, pO_2_ 52 mmHg). Upon imaging, consolidations were detected in the lower and middle lobes of the right lung as well as in the upper lobe of the left lung, which were initially interpreted as a result of a possible previous infection.

Due to severe dyspnea (RR 29/min) with persistent hypoxemia under low-flow oxygen and now manifest hypercapnic respiratory insufficiency (PaCO_2_ 66 mmHg, pH 7.23) high-flow oxygen therapy (HFNC) with intermittent non-invasive ventilation (NIV) was initiated. ECG showed sinus tachycardia (103 bpm) with normal axis. There was also no indication of IgG4-associated disease. There was no improvement with antibiotic therapy, so that under the suspicion of exacerbated inflammatory lung disease, steroid therapy (initially 1 g/day for 3 day followed by 1 mg/kg/day) was initiated along with diuretic therapy due to a tendency for peripheral edema in connection with known hypertensive heart disease. These measures led to an impressive improvement of the respiratory and clinical situation and normalization of blood gases when breathing room air; under these conditions, the 6-min walking distance was 400 m without ventilatory insufficiency or hypoxemia. The lung function test showed an obstructive respiratory disease (FEV1/FVC 56%, FEV1 57%-pred., Reff 128%-pred.), the diffusion capacity for carbon monoxide was reduced (53%-pred.).

**Figure 1:**
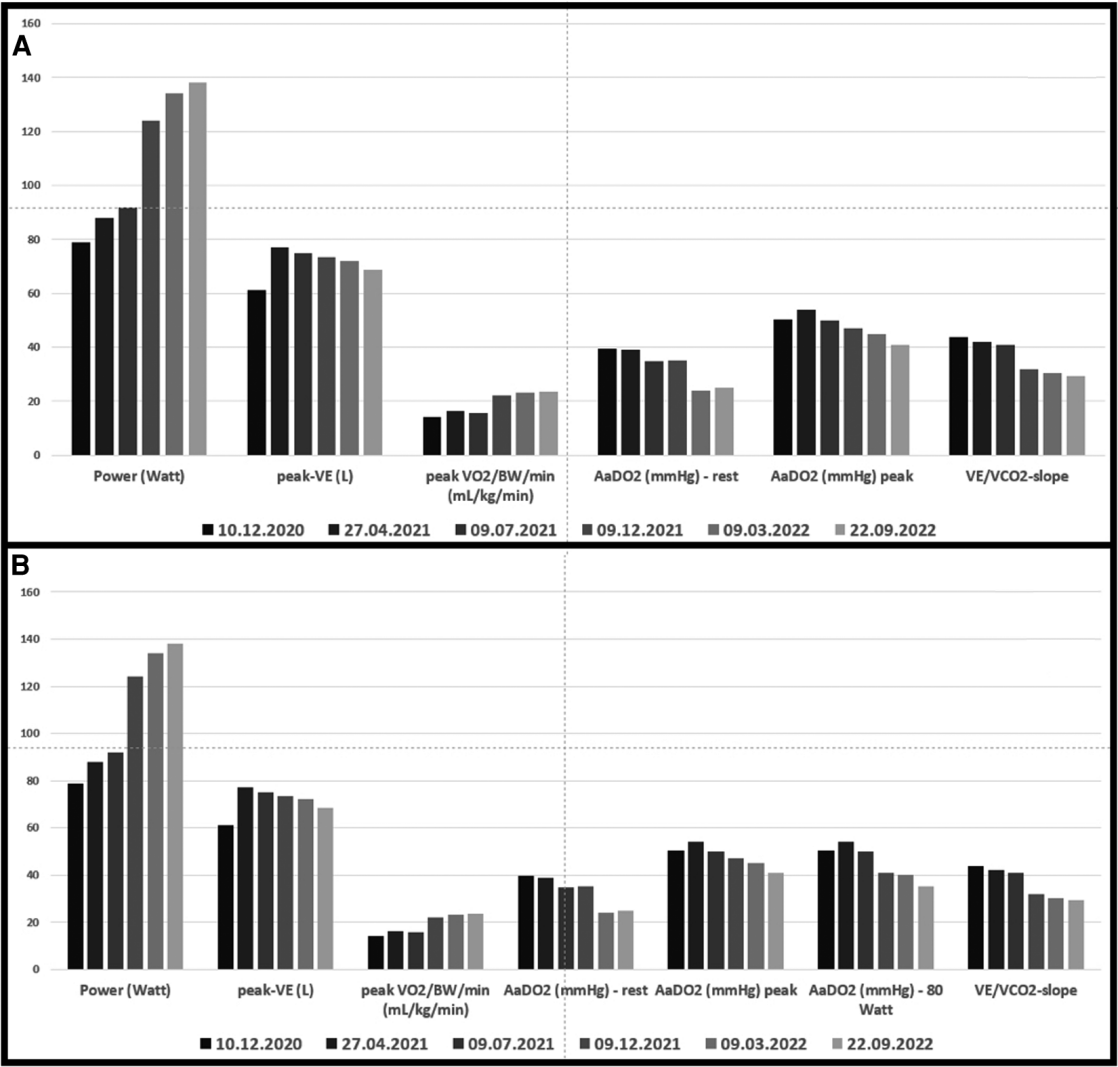
(**A**) Cardio-pulmonary exercise data. Grey bars before and green bars after intervention in 3/2021; (**B**) data from a) with the addition of AaDO_2_ at 80 Watt (maximal power achieved at the pre-interventional cardio-pulmonary exercise test).

In August 2020, a follow-up thoracic CT scan showed a complete regression of basal consolidations in the right lower lobe and clear regression of the remaining inflammatory changes. Steroid therapy was continued at 50 mg/day, reduced in dose over time, and discontinued in October 2020 (see timeline, Figure 1 and Table 1).

**Table 1:** Case reports without interventions.

	Total cases	Cases of isolated PV stenosis	Cases of isolated PA stenosis	Cases of PV and PA stenosis	Disease
Beaconsfield et al., 1998 (8)	*N* = 1		*N* = 1		Tuberculosis
Chang et al., 2012 (9)	*N* = 1			*N* = 1	Fibrosing Mediastinitis
Cimenoglu et al., 2019 (10)	*N* = 1		*N* = 1		Fibrosing Mediastinitis
Co et al., 2018 (4)	*N* = 1			*N* = 1	Fibrosing Mediastinitis
Cohen et al., 1996 (11)	*N* = 1		*N* = 1		Tuberculosis
Cosio et al., 1973 (12)	*N* = 1		*N* = 1		Fibrosing Mediastinitis
Damuth et al., 1980 (13)	*N* = 1		*N* = 1		Sarcoidosis
Gustafson et al., 2012 (14)	*N* = 1		*N* = 1		Fibrosing Mediastinitis (Histoplasmosis)
Hasegawa et al., 2012 (15)	*N* = 1		*N* = 1		Sarcoidosis
Kolbe et al., 1997 (16)	*N* = 1		*N* = 1		Fibrosing Mediastinitis
Lee et al., 2014 (17)	*N* = 1		*N* = 1		Fibrosing Mediastinitis
Leong et al., 2008 (18)	*N* = 1	*N* = 1			Fibrosing Mediastinitis
Li et al., 2018 (19)	*N* = 1	*N* = 1			Fibrosing Mediastinitis
Mahnken et al., 2001 (20)	*N* = 1	*N* = 1			Silicosis
Mangla et al., 1985 (21)	*N* = 1		*N* = 1		Sarcoidosis
Nelson et al., 1965 (22)	*N* = 1		*N* = 1		Fibrosing Mediastinitis
Ojeifo et al., 2015 (23)	*N* = 1		*N* = 1		Tuberculosis
Papandreou et al., 1992 (24)	*N* = 1		*N* = 1		Fibrosing Mediastinitis
Perez et al., 1999 (25)	*N* = 1		*N* = 1		Coccidioidomycosis
Songster et al., 2020 (26)	*N* = 1			*N* = 1	Fibrosing Mediastinitis (Histoplasmosis)
Yazaki et al., 2021 (27)	*N* = 1	*N* = 1			Anthracofibrosis
**Totals**	**21**	**4**	**14**	**3**	

## 3. Further diagnostic assessment, treatment, and clinical course

In December 2020, the patient again developed progressive exercise dyspnea with a productive cough and fatigue. Blood gas analysis showed hypoxemic respiratory insufficiency (pH 7,48, PaO_2_ 50 mmHg, PaCO_2_ 32 mmHg). Body plethysmography revealed an irreversible moderate obstructive ventilation disorder with reduced diffusion capacity (DLCO 60%-pred.).

**Figure 2:**
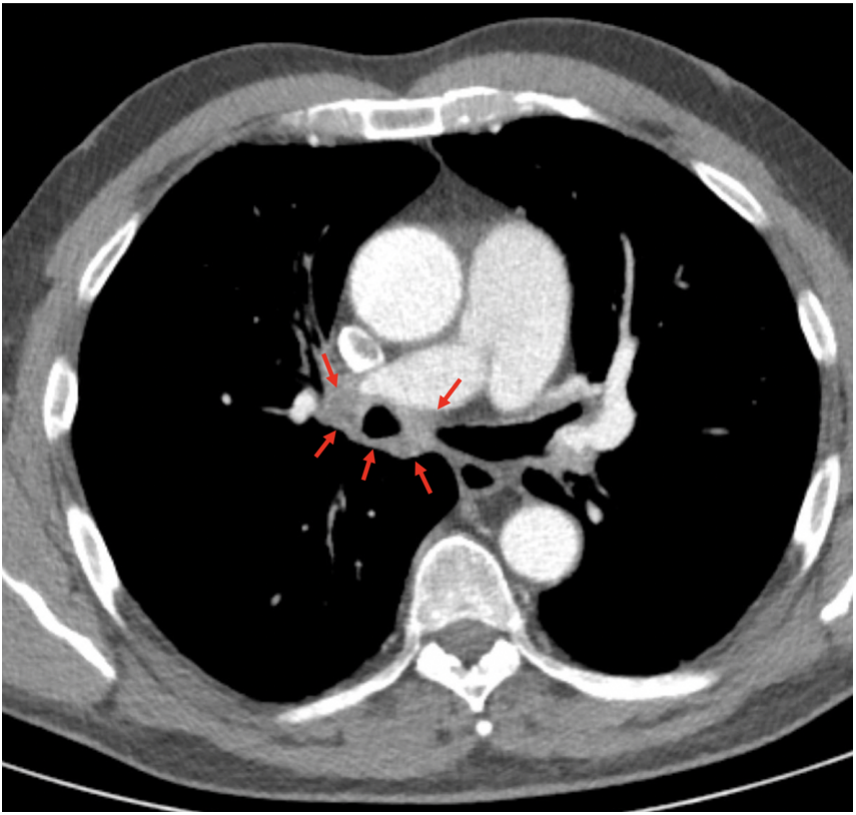
Preinterventional computed tomography showing perihilar soft tissue proliferation circumferentially located around the right main stem bronchus (red arrows).

Echocardiography revealed that systolic pulmonary arterial pressure was increased to 65 mmHg. Cardiopulmonary exercise testing (CPET) (Figure 1) showed a limited load (79 W, 60% of normal) as well as reduced ventilatory capacity, deconditioning, a pseudorestrictive breathing pattern, and abnormal diffusion with an AaDO_2_ of 50.3 mmHg, characteristic of pulmonary vasculopathy. Scintigraphy showed reduced perfusion in both lungs. There was no evidence of PA embolism in angio-CT scans of the thorax, but there was a compression of central regions of the left pulmonary arterial tree, with soft tissue proliferation on both sides, hilar and peribronchial (Figure 2), largely identical to the first imaging results from June 2020. Also striking was a compression-related concentric high-grade stenosis of the outflow portion of the left lower lobe artery, a moderate compression of the bronchus intermedius and the lower lobe bronchus, and a rupture of the middle lobe bronchus.

Rigid bronchoscopy with endobronchial ultrasound (EBUS) revealed extensive peribronchial compactions walling in surrounding tissue without recognizable boundaries as well as calcifications in the lymph nodes. Macroscopically, the biopsies were anthracotic. Histology revealed tumor-free lymph node particles with prominent anthracotic pigment and dust depositions. There were practically no positively labeled cells upon staining of MUM 1 as a marker of plasma cells or B cells. A conspicuous proliferation of IgG- or IgG4-positive plasma cells could be ruled out.

To further clarify the suspected vascular compression with consequent reduction of perfusion, selective pulmonary angiography supplemented by high-resolution angio-CT of the thorax was performed. The compression-related stenosis of the left lower lobe artery was confirmed, and stenosis of the right upper PV was also detected. Furthermore, a segmental pulmonary embolism was observed in the region of the right lower lobe. Moderate precapillary pulmonary hypertension with a PAm of 27 mmHg, pulmonary capillary wedge pressure of 4 mmHg, and a PVR of 4.4 WU was detected using right heart catheterization. Under load (40 W) there was a significant increase in PAm to 61 mmHg and in PVR to 5.1 WU.

**Figure 3:**
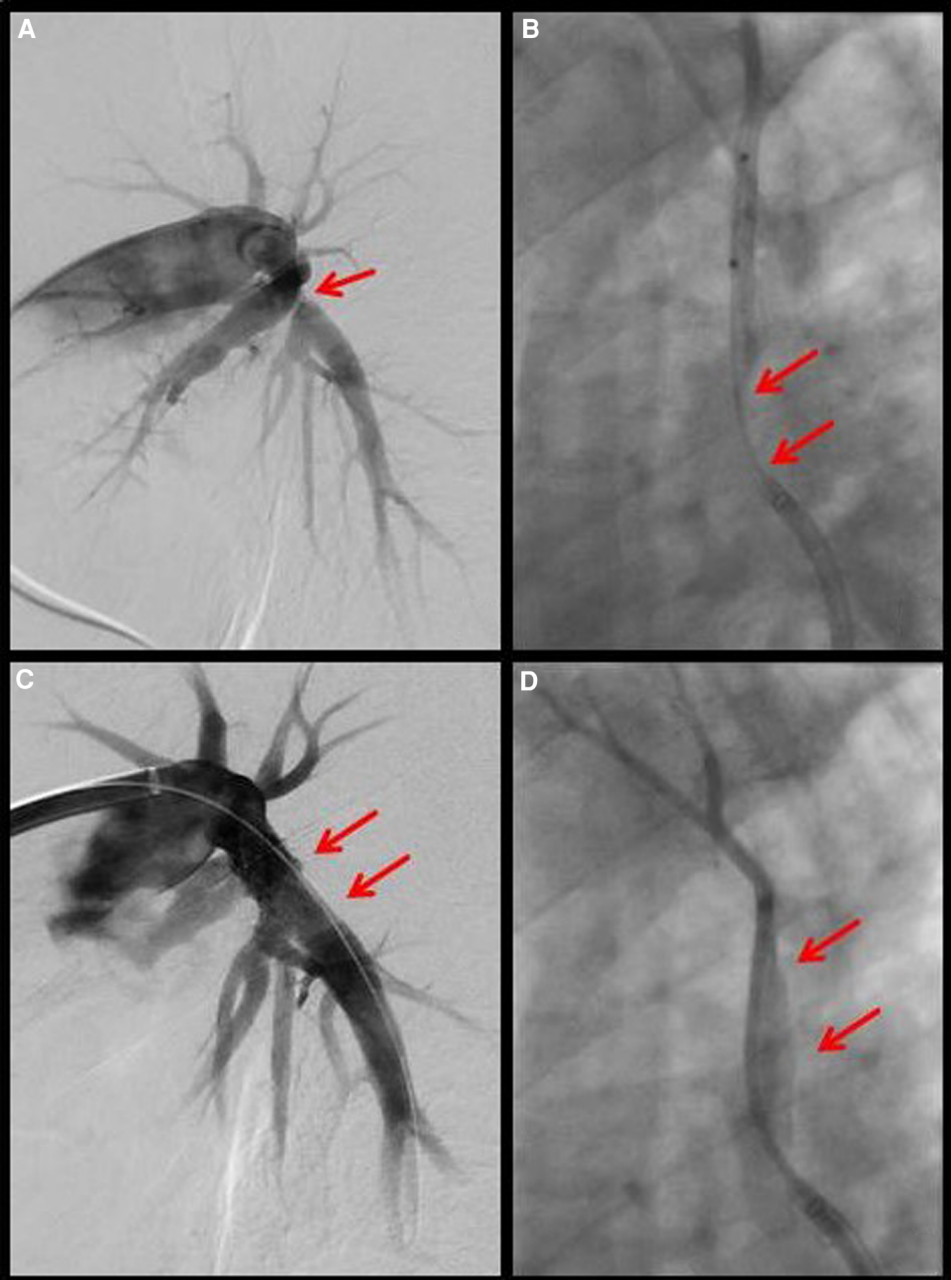
Intervention on the left interlobular pulmonary artery with high-grade stenosis before (**A**) and good flow after (**B**) stent insertion. Intervention on the highly stenosed upper right pulmonary vein (**C**) with good outflow after stent insertion (**D**).

Under renewed steroid therapy, simultaneous percutaneous intervention on the left interlobular PA and the right upper PV was carried out on March 11, 2021, by means of balloon dilatation and stent insertion (Figure 3): the left interlobar pulmonary artery was stented using an AndraTex Optimus CoCr Stent (L in 12 × 23 mm) after predilatation with Medtronic Admiral Xtreme 8.0 × 40 mm and 10.0 × 40 mm balloons. The right upper pulmonary vein was stented using a Cordis Palmaz Genesis (9 × 17 mm) after predilatation with a Cordis Powerflex Pro (6 × 20 mm). Post-procedural results were documented with selective angiography. Post-intervention the patient developed reperfusion edema of the left lower lobe of the lung that was quickly brought under control by NIV and negative fluid balance. A three-month dual anti-platelet therapy and oral anticoagulation (for 3 months at reduced dosage) were prescribed. Steroid therapy was gradually reduced to <5 mg/day. In the clinical course up to September 2022, CPET (Figure 1) showed increasing improvement in findings with significantly increased resilience (138 W, 92% normal), a decrease in diffusion disturbances, and an improved respiratory efficiency compared to the CPET before PA- and PV-stenting and the follow-up CPETs in 2021. Echocardiographically a normalization of PA pressure was seen.

## 4. Discussion

The present case report is the first description of simultaneous, successful stenting of concurrent stenosis of a pulmonary artery and vein with pulmonary hypertension in the context of mediastinal fibrosis with lymph node enlargement due to silicosis with anthracofibrosis. A similar stenting of pulmonary artery and vein stenosis in mediastinal fibrosis caused be Histoplasmosis was already described in 2001 by Doyle et al. (27). The diagnosis of vascular stenosis resulting from mediastinal fibrosis in the setting of silicosis is supported by the known occupational load, the bronchoscopically visible anthracotic mucosal changes, the CT findings with lymph node enlargement, including calcifications, and the histological changes with evidence of prominent anthracotic pigment and dust depositions as well as exclusion of other causes.

Initially, the pronounced clinical symptoms—swelling of the bronchial mucosa as is characteristic of anthracosilicosis, detection of infiltrates in the right lower lobe, and acute hypoxemic and hypercapnic insufficiency—were interpreted as signs of pneumonia complicated by cardiac decompensation with simultaneous pulmonary hypertension, especially since there was significant clinical improvement with diuretic therapy and non-invasive ventilation as well as complementary steroid therapy. Retrospectively, and with knowledge of the pulmonary vascular status, the transient respiratory deterioration and the pronounced bronchial mucosal thickening and bronchial stenosis can be explained by the impairment of pulmonary perfusion as well as intermittent pulmonary congestion as a result of PV stenosis. Similar findings for pulmonary hypertension, pulmonary venous congestion, and occasionally also additional pleural effusions and hemoptysis have already been described in connection with pulmonary vascular stenosis, mainly in the context of fibrosing mediastinitis (19, 20, 28–31). Wang et al. describe the dyad, triad, and tetralogy of pulmonary hypertension in fibrosing mediastinitis (PH-FM), which can even be seen in a chest radiograph. The FM dyad includes predominant main PA and lobe atelectasis. FM triad refers to the FM dyad plus pleural effusion or pulmonary congestion/interstitial pulmonary edema. FM tetralogy includes the mentioned 4 signs (9). Clinical indications of relevant stenosis of the pulmonary arteries were found in the CT of the thorax by the increasing vascular rarefication of the left lower lobe of the lung and the accordingly reduced perfusion in the scintigram. Pulmonary venous stenosis could only be diagnosed using pulmonary angiography and a supplementary high-resolution CT with appropriate contrast agent administration. This underlines the importance of employing high-quality and differential diagnostics for the assessment of pulmonary vascular involvement.

With differential diagnosis it is possible to essentially exclude fibrosing mediastinitis in undefined mediastinal processes. This is a rare disease caused by extensive fibroinflammatory changes with compression of vessels, especially the pulmonary vessels and the superior vena cava but also in individual cases the coronary vessels and esophagus (1, 2, 5, 15), that is characterized by a potentially lethal course. In most cases, fibrosing mediastinitis is infection associated (mostly histoplasmosis, occasionally in tuberculosis, coccidioidomycosis), non-infection associated (e.g., sarcoidosis, IgG4-related, silicosis), iatrogenic (e.g., radiotherapy, chest surgery) disease or it is idiopathic (3, 7, 9, 13, 15, 23, 32–34). In addition, further manifestations of multifocal fibrosis may be present (1).

The initially successful steroid therapy was suggestive of a steroid-sensitive, IgG4-associated disease; on the other hand, positive effects of steroids are also described that are independent of IgG4 (1, 2, 15). However, there were no clear indications, in particular serological or histological, in the present case. Here, the silicotic changes were particularly impressive.

In the present case, only the interventional treatment of vascular stenosis by means of stent implantation in the pulmonary artery and vein proved to be effective, both acutely and in the long term.

Besides echocardiography and B-type natriuretic peptide, CPET proved to be a sensitive non-invasive tool to detect functional impairment before stenting, as well as functional improvement after the intervention with normalization of parameters initially indicating pulmonary hypertension. Using these tools for surveillance, repeated angiographies may be avoided. However, suspicious findings and clinical deterioration should be clarified by imaging.

More than three decades ago, hybrid procedures with intraoperative stent insertion (23, 34–37) were performed in individual cases for the treatment of congenital PA and PV stenosis. Subsequently, interventional therapies in the form of balloon angioplasty and stenting were applied mainly for PV stenosis after ablation in atrial fibrillation (38, 39). In contrast, the clinical evidence on confirmed PA and PV stenosis in the context of fibrosing mediastinal processes is scarce, especially with regard to interventional therapy; there are primarily individual case reports, some with literature reviews (Tables 1, 2) (5, 19, 27, 29, 44). Fender et al. (5) describe in their overview several cases of successful percutaneous intervention but also refer to the limited experience with such procedures worldwide. In 2011 Albers et al. (7) reported a larger study of 58 patients with fibrosing mediastinitis, 46 of whom had PV and/or PA stenosis. In 53% of the cases, both vascular structures were involved. In addition, 12 patients had superior vena cava stenosis. Thirty patients were treated with multiple stenting of pulmonary veins and arteries. Reinterventions were required in five patients. In a retrospective analysis, Peikert et al. (3) observed a total of 80 cases of fibrosing mediastinitis between 1985 and 2006, 34 of which had vascular involvement. However, only 3 patients underwent angioplasty including stenting of the PA, while one patient received balloon dilatation of the PV. A more recent overview of 30 cases with PV dilatation and stent implantation was provided by Duan et al. (see also below) (42).

**Table 2:** Case reports with and without interventions (*16 cases without information about intervention).

	Total cases	Cases of isolated PV stenosis	Cases of isolated PA stenosis	Cases of PV and PA stenosis	Cases with dilatation ± stenting	PV stenosis, dilatation only	PV stenosis, dilatation and stenting	PA stenosis, dilatation only	PA stenosis, dilatation and stenting	Disease
Ahn et al., 2015 (40)	*N* = 1		*N* = 1		*N* = 1				*N* = 1	Silicosis
Albers et al., 2011 (7)	*N* = 46	n.a.	n.a.	*N* = 31	*N* = 30		*N* = 16		*N* = 19	Fibrosing Mediastinitis
Argueta et al., 2019 (33)	*N* = 2		*N* = 1		*N* = 1				*N* = 1	Fibrosing Mediastinitis (Coccidioidomycosis)
Condado et al., 2016 (41)	*N* = 5		*N* = 2	*N* = 3	*N* = 3		*N* = 1		*N* = 3	Sarcoidosis
Duan et al., 2022 (42)	*N* = 30	*N* = 30			*N* = 30		*N* = 30			Fibrosing Mediastinitis
Doucet et al., 2015 (43)	*N* = 1	*N* = 1			*N* = 1		*N* = 1			Fibrosing Mediastinitis (Histoplasmosis)
Doyle et al., 2001 (44)	*N* = 4	*N* = 2	*N* = 1	*N* = 1	*N* = 4		*N* = 3		*N* = 2	Fibrosing Mediastinitis
Ferguson et al., 2010 (45)	*N* = 4	*N* = 1	*N* = 3		*N* = 4		*N* = 1		*N* = 3	Fibrosing Mediastinitis
Guerrero et al., 2001 (46)	*N* = 1		*N* = 1		*N* = 1				*N* = 1	Fibrosing Mediastinitis
Li et al., 2020 (29)	*N* = 1		*N* = 1		*N* = 1				*N* = 1	Fibrosing Mediastinitis
Liu et al., 2015 (47)	*N* = 8		*N* = 8		*N* = 8				*N* = 8	Sarcoidosis
Ingraham et al., 2022 (6)	*N* = 1		*N* = 1		*N* = 1				*N* = 1	Fibrosing Mediastinitis
Kandzari et al., 2000 (48)	*N* = 1		*N* = 1		*N* = 1				*N* = 1	Fibrosing Mediastinitis
Peikert et al., 2011 (3)	*N* = 43	*N* = 10	*N* = 33		*N* = 4	*N* = 1			*N* = 3	Fibrosing Mediastinitis (incl. Histoplasmosis)
Ponamgi et al., 2015 (49)	*N* = 8	*N* = 8			*N* = 8	*N* = 3	*N* = 5			Fibrosing Mediastinitis
Satpathy et al., 2011 (50)	*N* = 1		*N* = 1		*N* = 1				*N* = 1	Fibrosing Mediastinitis (Histoplasmosis)
Seckeler et al., 2021 (32)	*N* = 1		*N* = 1		*N* = 1				*N* = 1	Fibrosing Mediastinitis (Coccidioidomycosis)
Shapiro et al., 2005 (51)	*N* = 1	*N* = 1			*N* = 1	*N* = 1				Fibrosing Mediastinitis
Smith et al., 2011 (52)	*N* = 1		*N* = 1		*N* = 1				*N* = 1	Fibrosing Mediastinitis
Thiessen et al., 2007 (53)	*N* = 1		*N* = 1		*N* = 1				*N* = 1	Fibrosing Mediastinitis
Unger et al., 2015 (30)	*N* = 1	*N* = 1			*N* = 1		*N* = 1			Fibrosing Mediastinitis
Valentin et al., 2016 (54)	*N* = 1		*N* = 1		*N* = 1				*N* = 1	Fibrosing Mediastinitis
Welby et al., 2021 (55)	*N* = 9		*N* = 9		*N* = 9				*N* = 9	Fibrosing Mediastinitis
Westhoff et al.,	*N* = 1	*N* = 1	*N* = 1	*N* = 1	*N* = 1		*N* = 1		*N* = 1	Silicosis
Yang et al., 2021 (56)	*N* = 1			*N* = 1	*N* = 1		*N* = 1		*N* = 1	Fibrosing Mediastinitis
Zhang et al., 2018 (28)	*N* = 1	*N* = 1			*N* = 1		*N* = 1			Silicosis, Anthracofibrosis
Zhou et al., 2019 (57)	*N* = 5	*N* = 5			*N* = 5		*N* = 5			Fibrosing Mediastinitis
**Totals**	**180** *	**61**	**68**	**35**	**122**	**5**	**66**	**0**	**60**	

In the context of silicosis, however, reports of PA or PV stenosis as a result of extensive fibrosis and/or lymph node enlargement (19, 20, 28) are rare. In a recent report, Yazaki et al. described a patient with dyspnea and pulmonary hypertension as a result of occlusion of the upper right PA (28). Similar to our case, the endoscopic imaging was characteristic of anthracofibrosis, and this diagnosis was supported by right heart catheterization and pulmonary angiography. The therapy administered with tadalafil seems rather unusual in view of the findings (28). Zhang et al. (20) reported for the first time in 2018 the successful percutaneous treatment of a case of PA stenosis with concomitant pulmonary hypertension in the context of mediastinal anthracofibrosis originating from silicosis. Here, pulmonary hypertension was particularly noteworthy. The definitive diagnosis could only be made by pulmonary angiography showing evidence of bilateral stenosis, which was successfully treated by balloon dilatation and stenting. Dilatation or stenting of stenosis of the pulmonary veins in silicosis and simultaneous arterial interventions have not yet been described.

A search of the literature produced a total of 120 cases of mediastinal fibrosis that were treated by dilatation of the pulmonary vessels, including 3 cases with both PA and PV stenosis (Table 2). In 71 of those 120 cases there was dilatation of PV stenoses with or without supplementary stenting, and in 60 cases PA dilatations were performed with or without stenting. In addition to the present case with simultaneous intervention, there is another in which both PA and PV stenting took place; however, this was a case of fibrosing mediastinitis (44) (Table 2).

The further clinical course after pulmonary vascular interventions shows a significant functional improvement in the majority of cases but can also be characterized by residual stenosis or restenosis. A number of studies provided detailed data about reduction of the degrees of stenosis, symptom relief, reduction of pulmonary pressure, re-stenosis, and survival. In the report by Albers et al. (7), 32 patients (87%), including patients with compression of the vena cava, experienced improvement in symptoms. This was also reflected in a significant decrease in pulmonary arterial pressures. There was a large variation in the period of persistent symptomatic improvement (2 to 144 months). Fifteen percent of the 40 patients treated by intervention died during follow-up. Five-year survival in patients with bilateral vascular involvement was significantly more favorable for patients with intervention than in those without (89.5% vs. 52.5%). In contrast, there was no significant difference between the two groups in cases of unilateral disease. In a case series described by Ponamgi et al. (49), there were 3 deaths among 8 patients with multiple dilated and stented pulmonary veins. Of 25 dilated veins, restenosis occurred in 11 during follow-up. Zhou et al. (57) reported in 2019 on 5 patients with fibrosing mediastinitis and PV stenosis treated by balloon dilatation and stent implantation. A total of 11 compression stenoses were treated without complications except for a cardiac arrest triggered by vagal stimulation. In the long-term course, one patient experienced renewed higher-grade stenosis at the proximal stent end as a result of fibrotic compression, while another patient developed in-stent thrombosis due to a cessation of anticoagulation. A re-evaluation at 6 months showed a significant improvement in the degree of stenosis and trans-stenotic pressure gradients in all cases. Pulmonary hypertension associated with pulmonary venous stenosis was improved post-interventionally, with a decrease in mean PA pressure from 45.0 ± 9.0 mmHg to 38.7 ± 8.4 mmHg (*p* < 0.05). Welby et al. (55) observed a significant increase of 54%–79% (*p* = 0.005) in lumen width after stenting of PA stenosis, with a simultaneous decrease in the stenosis gradient by an average of 9.38 mmHg (*p* = 0.005). The openness rate after one year was 90%, and 89% of patients reported an improvement in dyspnea. Re-interventions were required in 2 out of 9 patients. In the recent case study by Duan et al. (42) of 30 patients with fibrosing mediastinitis in the period 2018–2020, a total of 63 PV stenoses were treated in 32 angioplasty sessions and 44 stents were implanted in 41 pulmonary veins after prior balloon angioplasty. This strategy showed a significant improvement in clinical and functional findings in the WHO functional class with a simultaneous decrease in pulmonary arterial pressure by 5.4 mmHg, as was also observed in the present case. However, a significant number of post-interventional complications such as hemoptysis (18.8%) and lung injuries (44%) were described.

## 5. Conclusions

In summary, in cases of progressive dyspnea and concomitant mediastinal fibrosis, differential diagnostic clarification of the cause of fibrosis is required. In rare cases, this may be silicosis. Furthermore, especially in the presence of pulmonary hypertension, concomitant pulmonary vascular stenosis in the context of fibrosis must be taken into account. As in the current case, venous and arterial stenosis can be successfully treated by intervention. In the long term, this is reflected in a reduction in the extent of stenosis and pulmonary arterial pressures. In individual cases, however, residual stenosis may be present, or restenosis may occur.

## Data Availability

The data analyzed in this study is subject to the following licenses/restrictions: The authors obtained written informed consent from the patient for publication. However, requests to access these datasets should be directed to Michael Westhoff, michaelwesthoff.mail@t-online.de.

## References

[1] Westhoff M. Riedel-Struma und fibröse Mediastinitis. Ihre Beziehungen zur multifokalen Fibrose [Riedel's struma and fibrous mediastinitis. Their relation to multifocal fibrosis]. Dtsch Med Wochenschr. (1988) 113:348–51. 10.1055/s-2008-1067644 3278877

[2] Westhoff M. Riedel-Struma und fibröse Mediastinitis. Positive therapeutische Beeinflussbarkeit durch Corticoide. [Riedel's struma and fibrous mediastinitis. Positive therapeutic responsiveness to corticoids]. Dtsch Med Wochenschr. (1988) 113:337–41. 10.1055/s-2008-1067641 3345695

[3] Peikert T Colby TV Midthun DE Pairolero PC Edell ES Schroeder DR Fibrosing mediastinitis: clinical presentation, therapeutic outcomes, and adaptive immune response. Medicine (Baltimore). (2011) 90:411–23. 10.1097/MD.0b013e318237c8e6 22033450

[4] Co MLF Patel HN Agdamag AC Okwuosa TM. Fibrosing mediastinitis-related pulmonary artery and vein stenosis-limiting chemotherapy. BMJ Case Rep. (2018) 2018:bcr2017221905. 10.1136/bcr-2017-221905 29754128 PMC5950559

[5] Fender EA Widmer RJ Knavel Koepsel EM Welby JP Kern R Peikert T Catheter based treatments for fibrosing mediastinitis. Catheter Cardiovasc Interv. (2019) 94:878–85. 10.1002/ccd.28152 30790443

[6] Ingraham BS Packer DL Holmes DR Reddy YNV. The hemodynamic spectrum of pulmonary vein stenosis from fibrosing mediastinitis. Catheter Cardiovasc Interv. (2022) 99:198–200. 10.1002/ccd.29955 34536328

[7] Albers EL Pugh ME Hill KD Wang L Loyd JE Doyle TP. Percutaneous vascular stent implantation as treatment for central vascular obstruction due to fibrosing mediastinitis. Circulation. (2011) 123:1391–9. 10.1161/CIRCULATIONAHA.110.949180 21422386 PMC3095436

[8] Doyle TP Loyd JE Robbins IM. Case report: tuberculous pulmonary arteritis–an unusual cause of right pulmonary artery stenosis. Clin Radiol. (1998) 53:229–31. 10.1016/S0009-9260(98)80109-8 9528879

[9] Yazaki K Yoshida K Hyodo K Kanazawa J Saito T Hizawa N. Pulmonary hypertension caused by fibrosing mediastinitis. JACC Asia. (2022) 2:218–34. 10.1016/j.jacasi.2021.11.016 36338410 PMC9627819

[10] Zhang R Ma G Xu X Liang L. Pulmonary arterial bypass surgery for fibrosing mediastinitis causing severe pulmonary hypertension. Ann Thorac Surg. (2019) 107:e411–3. 10.1016/j.athoracsur.2018.09.067 30447188

[11] Mahnken AH Breuer C Haage P. Pulmonary artery reconstruction for tuberculosis. Ann Thorac Surg. (1996) 61:1257–9. 10.1016/0003-4975(95)01063-7 8607701

[12] Li Y Meng X Wang Y Yang Y Lu X. Pulmonary arterial stenosis with wide splitting of the second heart sound due to mediastinal fibrosis. Am J Cardiol. (1973) 31:372–6. 10.1016/0002-9149(73)90270-1 4569273

[13] Li YJ Pan X Wang C He B. Sarcoidosis complicated with major pulmonary artery obstruction and stenosis. Intern Med. (2012) 51:2775–80. 10.2169/internalmedicine.51.7693 23037473

[14] Gustafson MR Moulton MJ. Fibrosing mediastinitis with severe bilateral pulmonary artery narrowing: rV-RPA bypass with a homograft conduit. Tex Heart Inst J. (2012) 39:412–25. 22719157 PMC3368482

[15] Chang SH Shih CW Lei MH. Idiopathic mediastinal fibrosis with involvement of the pulmonary vessels and left main coronary artery. Catheter Cardiovasc Interv. (2012) 79:1019–22. 10.1002/ccd.23154 21542119

[16] Chang SH Shih CW Lei MH. Pulmonalarterienstenose bei aggressiver mediastinalfibrose; diagnostik und 3D-darstellung mittels helikaler CT-untersuchung. Aktuelle Radiol. (1997) 7:197–9. 9340017

[17] Lee CH Park JS. Pulmonary artery stenosis and aneurysm with idiopathic mediastinal fibrosis. Heart Lung Circ. (2014) 23:e190–191. 10.1016/j.hlc.2013.04.120 24931067

[18] Leong DP Dundon BK Steele PM. Unilateral pulmonary vein stenosis secondary to idiopathic fibrosing mediastinitis. Heart. (2008) 94:776. 10.1136/hrt.2007.124404 18480351

[19] Mahnken AH Breuer C Haage P. Silicosis-induced pulmonary artery stenosis: demonstration by MR angiography and perfusion MRI. Br J Radiol. (2001) 74:859–61. 10.1259/bjr.74.885.740859 11560837

[20] Zhang R Ma G Xu X Liang L. Percutaneous treatment for silicosis-induced pulmonary artery stenosis: a case report and review of the literature. Medicine (Baltimore). (2018) 97:e9469. 10.1097/MD.0000000000009469 29480834 PMC5943882

[21] Ojeifo O Gilotra NA Kemp CD Leventhal A Resar J Zehr KJ Sarcoidosis, pulmonary hypertension, and acquired peripheral pulmonary artery stenosis. Cathet Cardiovasc Diagn. (1985) 11:69–74. 10.1002/ccd.1810110110 3978707

[22] Nelson WP Lundberg GD Dickerson RB. Pulmonary artery obstruction and cor pulmonale due to chronic fibrous mediastinitis. Am J Med. (1965) 38:279–85. 10.1016/0002-9343(65)90182-8 14256724

[23] Massumi A Woods L Mullins CE Nasser WK Hall RJ. Successful management of fibrosing mediastinitis with severe vascular compromise: report of two cases and literature review. Respir Med Case Rep. (2019) 29:100987. 10.1016/j.rmcr.2019.100987 31890562 PMC6928374

[24] Papandreou L Panagou P Bouros D. Mediastinal fibrosis, and radiofrequency radiation exposure: is there an association? Respiration. (1992) 59:181–4. 10.1159/000196054 1439233

[25] Prieto LR Schoenhagen P Arruda MJ Natale A Worley SE. Pulmonary artery fibrous bands: report of a case with extensive lung infarction and superinfection with coccidioides immitis, Pseudomonas, and acid-fast bacilli. Arch Pathol Lab Med. (1999) 123:170–2. 10.5858/1999-123-0170-PAFB 10050795

[26] Neumann T Kuniss M Conradi G Sperzel J Berkowitsch A Zaltsberg S Pulmonary artery stenosis in a patient with prior histoplasmosis and the discovery of complications. J Cardiothorac Vasc Anesth. (2020) 34:832–4. 10.1053/j.jvca.2019.10.037 31767521

[27] Doyle TP Loyd JE Robbins IM. Percutaneous pulmonary artery and vein stenting: a novel treatment for mediastinal fibrosis. Am J Respir Crit Care Med. (2001) 164:657–60. 10.1164/ajrccm.164.4.2012132 11520733

[28] Duan YC Su HL Wei R Jiang KY Wang AQ Yang YH Pulmonary hypertension due to silicosis and right upper pulmonary artery occlusion with bronchial anthracofibrosis. Respir Med Case Rep. (2021) 34:101522. 10.1016/j.rmcr.2021.101522 34646731 PMC8497993

[29] Ponamgi SP DeSimone CV Lenz CJ Coylewright M Asirvatham SJ Holmes DR Fibrosing mediastinitis with pulmonary hypertension as a complication of pulmonary vein stenosis: a case report and review of the literature. Medicine (Baltimore). (2018) 97:e9694. 10.1097/MD.0000000000009694 29369193 PMC5794377

[30] Zhou X Li YJ Cao YS Su HL Duan YC Su X Successful stenting of bilateral pulmonary veins stenosis secondary to idiopathic fibrosing mediastinitis. JACC Cardiovasc Interv. (2020) 13:1003–5. 10.1016/j.jcin.2019.10.042 32007463

[31] Welby JP Fender EA Peikert T Holmes DR Jr Bjarnason H Knavel-Koepsel EM. Successful stenting of left pulmonary veins stenosis resulting from fibrosing mediastinitis. Eur Heart J. (2015) 36:2623. 10.1093/eurheartj/ehu511 25586119

[32] Beaconsfield T Newman-Sanders A Birch H Glenville B Al-Kutoubi A. Major pulmonary artery stenosis causing pulmonary hypertension in sarcoidosis. Chest. (1980) 78:888–91. 10.1378/chest.78.6.888 7449474

[33] Seckeler MD Pineda JRET Lotun K. Successful transcatheter recanalization of a chronically occluded left pulmonary artery due to fibrosing mediastinitis. JACC Cardiovasc Interv. (2021) 14:e215–216. 10.1016/j.jcin.2021.04.004 34147384

[34] Cohen AS Beaconsfield T al-Kutoubi A Handler CE Glenville BE. Mediastinal fibrosis of the pulmonary artery secondary to Tuberculosis. AnnThorac Surg. (2015) 100:e49–50. 10.1016/j.athoracsur.2015.03.113 26354666

[35] Cosio FG Gobel FL Harrington DP Sako Y. Intraoperative and percutaneous stenting of congenital pulmonary artery and vein stenosis. Circulation. (1993) 88:II210–217. 8222156

[36] Gustafson MR Moulton MJ. Pulmonary venous dilatation in pulmonary veno-occlusive disease. Am J Cardiol. (1981) 48:585–9. 10.1016/0002-9149(81)90092-8 7270465

[37] Kolbe M Helwig A Habicht JM Steinbrich W. Pulmonary artery stents: long-term follow-up. Catheter Cardiovasc Interv. (2010) 75:757–64. 10.1002/ccd.22356 20146310

[38] Lee CH Park JS. Comparison of stent versus balloon angioplasty for pulmonary vein stenosis complicating pulmonary vein isolation. J Cardiovasc Electrophysiol. (2008) 19:673–8. 10.1111/j.1540-8167.2008.01110.x 18284494

[39] Leong DP Dundon BK Steele PM. Pulmonary vein stenting for the treatment of acquired severe pulmonary vein stenosis after pulmonary vein isolation: clinical implications after long-term follow-up of 4 years. J Cardiovasc Electrophysiol. (2009) 20:251–7. 10.1111/j.1540-8167.2008.01316.x 19261037

[40] Mangla A Fisher J Libby DM Saddekni S. Percutaneous treatment for pulmonary hypertension caused by pulmonary artery stenosis due to anthracosis. Int J Tuberc Lung Dis. (2015) 19:747–8. 10.5588/ijtld.14.0787 25946371

[41] Nelson WP Lundberg GD Dickerson RB. Pulmonary stenting for the treatment of sarcoid induced pulmonary vascular stenosis. Sarcoidosis Vasc Diffuse Lung Dis. (2016) 33:281–7. 27758995

[42] Papandreou L Panagou P Bouros D. Short-term efficacy and perioperative safety of catheter-based intervention for pulmonary vein stenosis caused by fibrosing mediastinitis. Zhonghua Xin Xue Guan Bing Za Zhi. (2022) 50:55–61. 10.3760/cma.j.cn112148-20210507-00398 35045615

[43] Perez MT Alexis JB Ferreira T Garcia HP. Pulmonary hypertension due to fibrosing mediastinitis treated successfully with stenting of pulmonary vein stenoses. Can J Cardiol. (2015) 31:548.e5–7. 10.1016/j.cjca.2014.12.025 25840104

[44] Songster JC Liu H Brakke TR Aron RA. Pulmonary hypertension complicating fibrosing mediastinitis. Medicine (Baltimore). (2015) 94:e1800. 10.1097/MD.0000000000001800 26554778 PMC4915879

[45] Ahn JH Ahn JM Lee SW Choi SH Oh SY Lee SM Results of intravascular stent placement for fibrosing mediastinitis. Congenit Heart Dis. (2010) 5:124–33. 10.1111/j.1747-0803.2010.00387.x 20412484

[46] Condado JF Babaliaros V Henry TS Kaebnick B Kim D Staton GW Jr. Treatment of pulmonary artery compression due to fibrous mediastinitis with endovascular stent placement. Chest. (2001) 119:966–8. 10.1378/chest.119.3.966 11243985

[47] Doucet KM Labinaz M Chandy G Mielniczuk L Stewart D Contreras-Dominguez V Interventional therapy in sarcoidosis-associated pulmonary arterial stenosis and pulmonary hypertension. Clin Respir J. (2017) 11:906–14. 10.1111/crj.12435 26666961

[48] Ferguson ME Cabalka AK Cetta F Hagler DJ. Percutaneous stenting of right pulmonary artery stenosis in fibrosing mediastinitis. Catheter Cardiovasc Interv. (2000) 49:321–4. 10.1002/(SICI)1522-726X(200003)49:3<321::AID-CCD20>3.0.CO;2-5 10700067

[49] Guerrero A Hoffer EK Hudson L Schuler P Karmy-Jones R. Catheter-based intervention for pulmonary vein stenosis due to fibrosing mediastinitis: the mayo clinic experience. Int J Cardiol Heart Vascul. (2015) 8:103–7. 10.1016/j.ijcha.2015.06.005 PMC4765364 26925456

[50] Liu L Xu J Zhang Y Fang L Chai Y Niu M Fibrosing mediastinitis presenting as pulmonary stenosis: stenting works. Int J Cardiol. (2007) 118:e85 8–6. 10.1016/j.ijcard.2007.01.030 17399816

[51] Kandzari DE Warner JJ O'Laughlin MP Harrison JK. Cardiovascular collapse induced by position-dependent pulmonary vein occlusion in a patient with fibrosing mediastinitis. Anesthesiology. (2005) 103:661–3. 10.1097/00000542-200509000-00032 16129994

[52] Satpathy R Aguila V Mohiuddin SM Khan IA. Pulmonary artery stenosis secondary to fibrosing mediastinitis: management with cutting balloon angioplasty and endovascular stenting. Vasc Endovascular Surg. (2011) 45:170–3. 10.1177/1538574410393034 21278182

[53] Shapiro BP Sprung J Scott K Arendt K Krowka MJ Afessa B. Fibrosing mediastinitis: successful stenting of the pulmonary artery. Can Respir J. (2008) 15:41–4. 10.1155/2008/835921 18292853 PMC2677855

[54] Smith JS Kadiev S Diaz P Cheatham J. Endovascular treatment of bilateral pulmonary artery stenoses and superior vena Cava syndrome in a patient with advanced mediastinal fibrosis. Tex Heart Inst J. (2016) 43:249–57. 10.14503/THIJ-15-5091 27303243 PMC4894706

[55] Thiessen R Matzinger FR Seely J Aina R Macleod P. Evaluation of outcomes following pulmonary artery stenting in fibrosing mediastinitis. Cardiovasc Intervent Radiol. (2021) 44:384–91. 10.1007/s00270-020-02714-z 33205295

[56] Valentin LI Kuban JD Ramanathan R Whigham CJ. Refractory pleural effusion as a rare complication of pulmonary vascular stenosis induced by fibrosing mediastinitis: a case report and literature review. J Int Med Res. (2021) 49:3000605211010073. 10.1177/03000605211010073 33947262 PMC8113940

[57] Yang S Wang J Li J Huang K Yang Y. Feasibility and efficacy of percutaneous pulmonary vein stenting for the treatment of patients with severe pulmonary vein stenosis due to fibrosing mediastinitis. Zhonghua Xin Xue Guan Bing Za Zhi. (2019) 47:814–81. 10.3760/cma.j.issn.0253-3758.2019.10.008 31648464

